# Efficacy of different nonsurgical treatments for peri-implantitis: a multi-arm randomized controlled clinical trial

**DOI:** 10.1038/s41598-026-50332-5

**Published:** 2026-05-02

**Authors:** Nihal Eraydin-Tufek, Gonen Ozcan, Sila Cagri Isler

**Affiliations:** 1 Department of Periodontology, Faculty of Dentistry, Yeditepe University, Istanbul, Turkey; 2 Department of Periodontology, Faculty of Dentistry, Gazi University, Ankara, Turkey

**Keywords:** Peri-implantitis, Nonsurgical treatment, Implant decontamination, Glycine powder air abrasion, Chlorhexidine, Ozone therapy, Diseases, Health care, Medical research

## Abstract

Peri-implantitis remains a challenging biofilm-associated condition, and the comparative effectiveness of adjunctive nonsurgical decontamination approaches is still unclear. This randomized, assessor-blinded, multi-arm clinical trial evaluated the clinical performance of different local submarginal decontamination protocols used alone or in combination with mechanical instrumentation in the management of early peri-implantitis lesions. Eighty implants from 26 patients were allocated to five treatment groups: mechanical instrumentation alone, mechanical instrumentation combined with chlorhexidine irrigation, ozone application, or glycine powder air abrasion, and glycine powder air abrasion as monotherapy. Clinical parameters, including probing pocket depth, bleeding on probing, and modified plaque index, were assessed at baseline, 3 months, and 6 months, and analyzed using linear mixed-effects models accounting for clustering at the patient level. All treatment modalities resulted in significant clinical improvements over 6 months. The greatest numerical reductions in probing depth, bleeding on probing, and plaque index were observed when mechanical instrumentation was combined with glycine powder air abrasion; however, no statistically significant differences were detected among treatment groups. These findings suggest that nonsurgical mechanical instrumentation, with or without adjunctive approaches, can provide meaningful short-term improvements in early peri-implantitis, while adjunctive glycine powder air abrasion may offer additional clinical benefits without demonstrating clear overall superiority.

## Introduction

Peri-implantitis is a biofilm-associated pathological condition characterized by mucosal inflammation and progressive loss of supporting tissues^1,2^. Although prevalence estimates vary, numerous studies highlight its high global burden^3–5^. Given its strong link to bacterial biofilm, contemporary management primarily targets biofilm control and inflammation resolution. In line with the EFP clinical practice guideline for peri-implant therapy, professional mechanical plaque removal (PMPR) represents a fundamental component of nonsurgical peri-implantitis care, supporting inflammation control and long-term stability. Management of peri-implantitis generally follows a stepwise approach, beginning with nonsurgical therapy, including PMPR, and escalating to surgical intervention where indicated^6^. However, mechanical submarginal instrumentation alone provides only modest yet measurable benefits in early lesions, typically reducing bleeding tendency (20–50%) and probing depth by ≤ 1 mm^7^. Decontamination may be further limited by implant morphology, surface characteristics, and prosthetic design. Consequently, several adjunctive or alternative interventions—including antibiotics, antiseptics, lasers, ozone, air abrasion systems, and air abrasion with glycine or erythritol powders—have been proposed to enhance surface decontamination and improve clinical outcomes^8^.

Among these modalities, adjunctive chlorhexidine digluconate (CHX) has shown reductions in inflammatory parameters^9–11^, whereas ozone—despite limited evidence^12^—may promote healing through potent antimicrobial activity^13^. Glycine powder air abrasion has also been used either as an adjunct or monotherapy^9,14–16^. While nonsurgical peri-implant therapy, with or without adjunctive measures, tends to reduce probing pocket depth (PPD) and bleeding on probing (BoP), current evidence does not indicate clear superiority of any specific approach^17–19^.

Therefore, the aim of the present study was to evaluate the clinical effectiveness of different local submarginal decontaminating measures used either alone or in combination with adjunctive therapies, including chlorhexidine digluconate (CHX) irrigation, gaseous ozone application, or glycine powder air abrasion, as well as glycine powder air abrasion used as monotherapy, in the management of early-staged peri-implantitis lesions.

## Methods

### Study design and participants

This was a prospective, randomized, multi-arm clinical trial with five parallel treatment groups, conducted between November 2017 and June 2018, with 6-month follow-up. This multi-arm parallel-group randomized clinical trial was reported in accordance with the CONSORT 2010 statement and the expanded CONSORT guidance for multi-arm clinical trials^20^. The protocol received ethics approval before enrolment (Ankara University Faculty of Dentistry, Ref:36290600/118; Turkish Medicines and Medical Devices Agency, Ref:71146310 2017–076). This trial was retrospectively registered at ClinicalTrials.gov (NCT06730568; 09/12/2024) in accordance with ICMJE transparency requirements.

The participants were recruited from patients who had previously received implant therapy at the Faculty of Dentistry, Gazi University and later presented for follow-up or peri-implant-related complaints. Patients exhibiting clinical signs of inflammation, such as increased PPD and BoP, along with radiographic evidence of peri-implant bone loss compared to baseline or previous follow-up records, were considered to have peri-implantitis and were examined for eligibility according to the inclusion criteria. Implants were classified as early peri-implantitis according to the clinical severity-based classification proposed by Froum and Rosen^21^, defined as probing depths of 4–6 mm combined with bleeding on probing and radiographic bone loss < 25% of the implant length. The study was conducted as a single-center trial, and data collection and analysis were also performed at the same center.

The study population consisted of 26 partially or fully edentulous patients diagnosed with early peri-implantitis, with a total of 80 implants that fulfilled the inclusion criteria. Written informed consent was obtained from all participants.

The following inclusion criteria were used for the selection of patients: (a) the presence of at least one titanium implant with clinical and radiographic signs of early peri-implantitis^21,22^; (b) cemented or screw type restorations without overhangs; (c) absence of occlusal overload; (d) the presence of at least 2 mm of keratinized attached peri-implant mucosa; (e) healthy or treated periodontitis and proper periodontal maintenance care; and (f) a good level of oral hygiene (full mouth plaque score ≤ 25%). The exclusion criteria were as follows: (a) the presence of implant mobility, (b) systemic diseases (i.e., diabetes (HbA1c ≥ 7%), osteoporosis, bisphosphonate medication) that could affect the outcome of treatment, (c) smoking six or more cigarettes per day, and (d) implants placed and prosthetically loaded within the previous 12 months.

### Randomization, allocation concealment and blinding

Randomization was carried out at the implant level, with all implants within the same patient allocated to the same treatment group, thereby preventing within-patient cross-treatment contamination via a computer-generated random number sequence to ensure equal allocation of 16 implants per group. Allocation concealment was maintained through the use of sealed, opaque, sequentially numbered envelopes containing group assignment codes. These envelopes were opened by the operator immediately prior to the intervention, following the administration of local anesthesia, and the corresponding treatment was performed on the assigned implant.

The participants were randomly assigned to one of five groups:

(i) Control group (M): mechanical instrumentation with titanium curettes (ImplaMate, Nordent Manufacturing Inc., Elk Grove Village, IL, USA);
(ii) Chlorhexidine group (M-CHX): mechanical instrumentation with titanium curettes followed by 0.2% CHX irrigation (Klorhex^®^ 0.2%, DROGSAN, Ankara, Türkiye);
(iii) Ozone group (M-O): mechanical instrumentation with titanium curettes followed by gaseous ozone application (OzoneDTA^®^ Ozone Generator, Denta Tec Dental AS, Hov, Norway);
(iv) Glycine powder air abrasion group (M-GPA): mechanical instrumentation with titanium curettes followed by glycine powder air abrasion (AIR-FLOW^®^ handy 3.0 PERIO^®^, EMS, Nyon, Switzerland);
(v) GPA: Glycine powder air abrasion monotherapy.

All treatment procedures were performed by a single trained and calibrated operator (N.E-T.), who had undergone standardization procedures prior to the study. All clinical measurements were conducted by an experienced and calibrated examiner (S.C.I.), with demonstrated intra-examiner reliability (κ > 0.80), who remained blinded to the group allocation throughout the study. Due to the nature of the interventions, the treating operator and the participants could not be blinded to the assigned treatment.

The null hypothesis (H0) stated that there would be no statistically significant between-group difference in mean PPD change following mechanical instrumentation alone, mechanical instrumentation plus CHX, mechanical instrumentation plus gaseous ozone, mechanical instrumentation plus glycine air abrasion, or glycine air abrasion alone.

### Interventions

The implant-supported prostheses were not removed during the clinical measurements or interventions.

In the control group M, mechanical submarginal instrumentation was performed at the peri-implantitis sites via titanium curettes. Instrumentation was continued until the operator determined that the implant surface was adequately debrided.

In the M-CHX group, adjunctive 0.2% CHX irrigation was performed immediately after mechanical submarginal instrumentation with titanium curettes.

In the M-O test group, adjunctive gaseous ozone was applied immediately after mechanical submarginal instrumentation with titanium curettes. A fixed concentration of 2100 ppm ozone, with 80% oxygen, was delivered to four sites (mesial, distal, buccal, and lingual) for 15 s per site via ozone generator device equipped with a periodontal tip (PA Probe^®^, Ozone Generator, Denta Tec Dental AS, Hov, Norway), in accordance with the manufacturer’s instructions.

In the test group M-GPA, adjunctive air abrasion therapy with glycine powder (AIR-FLOW^®^ HANDY 3.0 with PERIOFLOW^®^ nozzle; Powder PERIO^®^, EMS, Nyon, Switzerland) was applied immediately after mechanical submarginal instrumentation with titanium curettes. The procedure was performed via a flexible, calibrated perio-tip nozzle inserted into the peri-implant pockets at four sites (mesial, distal, buccal, and lingual) for 5 s per site. The handpiece was guided in a coronoapical direction with a circular, noncontact motion parallel to the implant surface, in accordance with the manufacturer’s instructions.

In test Group GPA, the peri-implantitis sites were treated with the same air abrasion system and glycine powder, which were applied as a monotherapy without prior mechanical instrumentation.

### Supportive periodontal/peri-implant care

All patients were enrolled in an individualized oral hygiene program at least 4 weeks prior to treatment. Oral hygiene instructions were provided according to the specific needs of each patient. Hard and soft deposits on the supramarginal surfaces of both the implants and teeth were removed. Oral hygiene maintenance was provided at baseline and at weeks 4, 8, 12, 16, 20, and 24.

### Clinical outcomes

The primary outcome of the study was to evaluate differences in mean PPD among the control and test groups after 6 months of nonsurgical therapy. Secondary outcomes included changes in BoP and mPI, as well as the proportion of implants achieving treatment success, defined as the absence of PPD ≥ 5 mm, absence of BoP/suppuration or the presence of BoP at no more than one site, and no additional radiographic bone loss.

Clinical measurements were performed via a color-coded University of North Carolina periodontal probe (NORDENT Manufacturing Inc., Elk Grove Village, IL, USA) with a standardized probing force of 0.2 N at baseline (T0), 3 months (T1), and 6 months (T2).

PPD was measured to the nearest millimeter from the mucosal margin to the bottom of the pocket. BoP was recorded as positive if bleeding occurred within 30 s of stimulation and negative if no bleeding was observed. mPI was scored on a scale from 0 to 3 based on the amount of plaque detected by gently running the probe across the smooth marginal surface of the implant^23^. Measurements were taken at four sites per implant (mesiobuccal, mid-buccal, distobuccal, and mid-lingual/palatal) by a single blinded examiner (S.C.I.). For analysis, site-level measurements were aggregated to the implant level as follows: PPD and mPI were averaged, and BoP was calculated as the percentage of bleeding sites.

### Statistical analysis and power calculation

The a priori sample-size calculation targeted detection of a 1.0-mm between-group difference in mean PPD change at 6 months (α = 0.05, 80% power) at the implant level, assuming independence between implants. No inflation was applied for intra-patient clustering or for multiplicity arising from the five-arm design. Accordingly, the study was powered for the primary continuous outcome only; analyses of treatment success were secondary and not formally powered.

All analyses were performed in R (R Core Team, 2024). To account for clustering of multiple implants within patients, mixed-effects models with a random intercept for patient were used.

For continuous outcomes (PPD, BoP, mPI), longitudinal change within each treatment arm was evaluated by fitting group-specific linear mixed-effects models with time (T0, T1, T2) as a fixed effect; pairwise contrasts (T0 vs T1; T0 vs T2) were derived from estimated marginal means with Holm–Bonferroni correction for multiple testing. Between-group differences at each follow-up (T1, T2) were assessed using linear mixed-effects models with group as a fixed effect, fitted separately at each time point. The overall group effect was tested by likelihood-ratio comparison of models with and without the group term; Holm-adjusted pairwise comparisons were performed conditional on a significant global test. Model assumptions were assessed by inspection of residuals. Two-sided *p* < 0.05 was considered statistically significant.

For the binary composite endpoint treatment success (PPD ≤ 5 mm and BoP at no more than one site per implant), generalized linear mixed-effects models (logit link) were fitted at the implant level with a random intercept for patient. Models were fit separately at T1 and T2 with treatment group as a fixed effect (mechanical instrumentation, M, as the reference). Odds ratios (OR) with 95% confidence intervals and Wald *p*-values were reported, and the overall group effect at each time point was assessed using a likelihood-ratio test. When the global test was significant, Holm–Bonferroni–adjusted pairwise comparisons were conducted. Marginal (model-based) predicted probabilities of success per group were obtained by marginal standardization from the fitted models. The intraclass correlation coefficient (ICC) was derived from the random-intercept variance to quantify clustering.

No imputation was required because there were no losses to follow-up; all randomized implants were analyzed at each time point.

No generative AI tools were used in the design, analysis, or writing of this manuscript.

## Results

### Study population

**Fig. 1:**
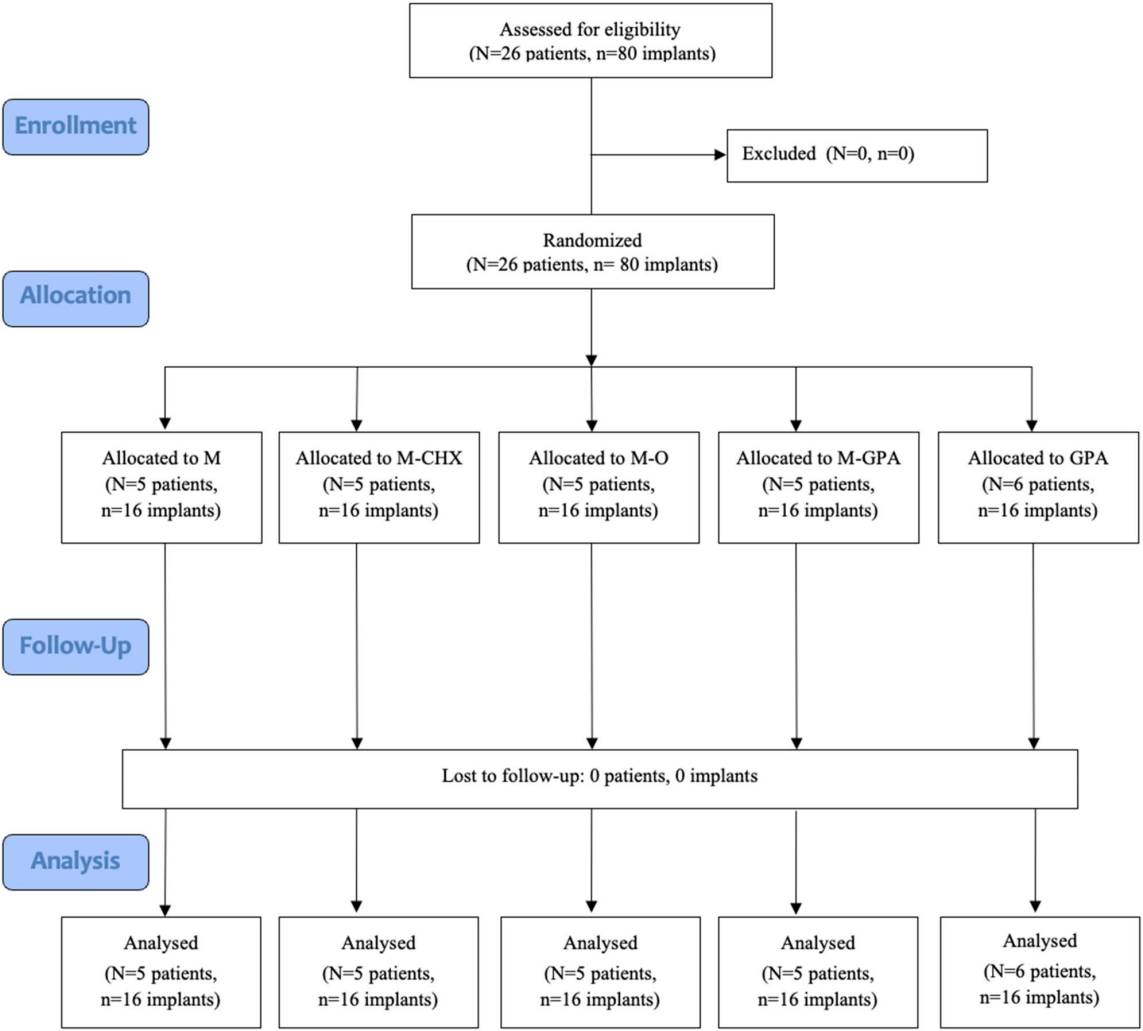
Consort flow diagram.

A total of 80 implants from 26 patients (12 males and 14 females, with mean ages of 53.7 ± 3.1 and 50.7 ± 2.7 years, respectively) met the inclusion criteria and were analyzed. The participants were evenly distributed across five groups, with equal allocation at the implant level. The number of patients per group was 5 or 6, resulting in an average of 3.08 implants per patient. All participants completed the study, and no adverse effects or dropouts were reported during the study period. The study flow chart is presented in Fig. 1.

### Demographic variables

The number of participants (N), number of implants (n), and mean age (years) in each group were as follows: M (N = 5, n = 16, 54.2 ± 7.6), M-CHX (N = 5, n = 16, 56.0 ± 11.2), M-O (N = 5, n = 16, 55.4 ± 3.6), M-GPA (N = 5, n = 16, 55.2 ± 13.5), and GPA (N = 6, n = 16, 41.7 ± 7.9). Detailed baseline demographic and implant-related data are presented in Table 1.

**Table 1:** Patient and implant characteristics at baseline.

Characteristics	M	M-CHX	M-O	M-GPA	GPA
Age (years- mean (SD))	54.2 (7.6)	56 (11.2)	55.4 (3.6)	55.2 (13.5)	41.7 (7.9)
Gender (n subjects (%))					
Male	3 (60)	1 (20)	3 (60)	3 (60)	2 (33.3)
Female	2 (40)	4 (80)	2 (40)	2 (40)	4 (66.7)
History of Periodontitis (n subjects (%))	5 (100)	5 (100)	3 (60)	3 (60)	3 (50)
Smoking (n subjects (%))					
Light^a^	2 (40)	1 (20)	1 (20)	1 (20)	1 (16.7)
Former	1 (20)	0 (0)	1 (20)	0 (0)	0 (0)
Never	2 (40)	4 (80)	3 (60)	4 (80)	5 (83.3)
Implant location (n implants (%))					
Maxilla	12 (75)	12 (75)	9 (56.3)	14 (87.5)	8 (50)
Mandible	4 (25)	4 (25)	7 (43.7)	2 (12.5)	8 (50)
Years in function (n implants (%))					
< 5	9 (56.3)	10 (62.5)	2 (12.5)	14 (87.5)	6 (37.5)
5–10	7 (43.8)	4 (25)	14 (87.5)	2 (12.5)	8 (50)
> 10	0 (0)	2 (12.5)	0 (0)	0 (0)	2 (12.5)
Brand					
AB		2 (12.5)	2 (12.5)		
Adin		2 (12.5)	2 (12.5)	3 (18.8)	7 (43.8)
Alpha Biotec	7 (43.8)	1 (6.3)			
Astra				2 (12.5)	
Nobel		2 (12.5)	4 (25)	9 (56.3)	7 (43.8)
Straumann	8 (50)	3 (18.8)	2 (12.5)	2 (12.5)	
Trias	1 (6.3)		6 (37.5)		
Xive		6 (37.5)			1 (6.3)
Zimmer					1 (6.3)

Values are presented as mean (standard deviation) for age and as number of subjects (percentage) for gender, history of periodontitis, and smoking status. Implant location and years in function are expressed at the implant level as number of implants (percentage).

^a^Light smokers were defined as patients who smoked fewer than 5 cigarettes per day.

### Implant level analysis

Implant-level clinical outcomes at baseline (T0), 3 months (T1), and 6 months (T2) are presented in Table 2. At baseline, no statistically significant differences were observed among the groups in mean PPD (χ^2^ = 3.49, df = 4, *p* = 0.479), BoP (χ^2^ = 4.27, df = 4, *p* = 0.370), or mPI (χ^2^ = 1.31, df = 4, *p* = 0.859), confirming the comparability of groups prior to intervention.

**Table 2:** Implant-level clinical outcomes at baseline (T0), 3 months (T1), and 6 months (T2) in the treatment groups.

	Time	Mean ± SD	Median	95% CI	*p*-value
PPD (mm)					
M	T0	4.95 ± 0.58	4.88	4.67–5.24	–
	T1	4.45 ± 0.60	4.63	4.16–4.75	
	T2	3.88 ± 0.61	4.00	3.57–4.18	
M-CHX	T0	4.69 ± 0.63	4.88	4.38–5.00	0.071
	T1	4.23 ± 0.61	4.50	3.94–4.53	
	T2	3.52 ± 0.73	3.50	3.16–3.87	
M-O	T0	4.58 ± 0.58	4.50	4.29–4.86	0.507
	T1	3.98 ± 0.59	4.00	3.70–4.27	
	T2	3.70 ± 0.53	3.63	3.45–3.96	
M-GPA	T0	4.75 ± 0.52	4.75	4.50–5.00	0.334
	T1	4.05 ± 0.68	4.13	3.71–4.38	
	T2	3.23 ± 0.52	3.38	2.98–3.49	
GPA	T0	4.45 ± 0.76	4.50	4.08–4.83	0.193
	T1	4.23 ± 0.67	4.25	3.91–4.56	
	T2	3.89 ± 0.58	3.88	3.60–4.18	
BoP (%)					
M	T0	67.19 ± 38.43	75.00	48.36–86.02	–
	T1	48.44 ± 32.23	62.50	32.64–64.23	
	T2	26.56 ± 23.22	25.00	15.19–37.94	
M-CHX	T0	73.44 ± 28.09	75.00	59.67–87.20	0.213
	T1	46.88 ± 25.62	50.00	34.32–59.43	
	T2	21.88 ± 20.16	25.00	12.00–31.75	
M-O	T0	65.62 ± 30.10	75.00	50.87–80.38	0.232
	T1	34.38 ± 35.21	25.00	17.12–51.63	
	T2	26.56 ± 23.22	25.00	15.19–37.94	
M-GPA	T0	82.81 ± 28.46	100.00	68.87–96.76	0.841
	T1	32.81 ± 28.46	25.00	18.87–46.76	
	T2	9.38 ± 17.97	0.00	0.57–18.18	
GPA	T0	81.25 ± 21.41	87.50	70.76–91.74	0.862
	T1	67.19 ± 19.83	62.50	57.47–76.90	
	T2	35.94 ± 31.58	25.00	20.46–51.41	
mPI					
M	T0	1.19 ± 0.35	1.00	1.02–1.36	–
	T1	0.86 ± 0.43	0.75	0.65–1.07	
	T2	0.50 ± 0.42	0.38	0.30–0.70	
M-CHX	T0	0.98 ± 0.23	1.00	0.87–1.10	0.351
	T1	0.45 ± 0.31	0.50	0.30–0.60	
	T2	0.33 ± 0.37	0.25	0.15–0.51	
M-O	T0	1.17 ± 0.22	1.25	1.06–1.28	0.836
	T1	0.80 ± 0.39	1.00	0.61–0.99	
	T2	0.48 ± 0.39	0.50	0.29–0.68	
M-GPA	T0	1.16 ± 0.24	1.00	1.04–1.27	0.150
	T1	0.48 ± 0.30	0.50	0.34–0.63	
	T2	0.23 ± 0.32	0.00	0.08–0.39	
GPA	T0	1.33 ± 0.51	1.00	1.08–1.58	0.885
	T1	0.94 ± 0.38	1.00	0.75–1.12	
	T2	0.66 ± 0.30	0.75	0.51–0.80	

Mean values ± standard deviations and medians of probing pocket depth (PPD, mm), bleeding on probing (BoP, %), and modified plaque index (mPI) are presented for each time point. CI: confidence interval.

### Intragroup changes over time

Overall, intragroup analyses demonstrated significant clinical improvements across most parameters.

- Group M: Significant reductions were observed at both T1 and T2 for all parameters (PPD: *p* = 0.006, *p* < 0.001; BoP: *p* = 0.029, *p* < 0.001; mPI: *p* = 0.009, *p* < 0.001).
- Group M-CHX: All parameters improved significantly by T2 (all *p* < 0.001). Between T0 and T1, reductions in BoP and mPI were significant (both *p* < 0.001), whereas the decrease in PPD showed a marginal trend (*p* = 0.051).
- Group M-O: PPD, BoP, and mPI decreased significantly at both follow-ups (PPD: *p* = 0.001, *p* < 0.001; BoP: *p* = 0.001, *p* < 0.001; mPI: both *p* < 0.001).
- Group M-GPA: All clinical parameters exhibited significant and consistent reductions at T1 and T2 (all *p* < 0.001).
- Group GPA: Significant reductions in PPD and BoP were observed only at T2 (PPD: *p* = 0.007; BoP: *p* < 0.001), while changes at T1 were not significant (PPD: *p* = 0.292; BoP: *p* = 0.084). In contrast, mPI improved significantly at both T1 and T2 (*p* = 0.004, *p* < 0.001).

### Intergroup comparisons

Intergroup comparisons of changes in clinical parameters are summarized in Table 3.

**Table 3:** Changes in clinical parameters (Δ) between time intervals (T0–T1, T1–T2, and T0–T2) at the implant level in the treatment groups.

	Δ T0–T1 Mean ± SD (Median)	95% CI	Δ T1–T2 Mean ± SD (Median)	95% CI	Δ T0–T2 Mean ± SD (Median)	95% CI
PPD (mm)						
M	0.50 ± 0.77 (0.38)	0.12–0.88	0.58 ± 0.52 (0.63)	0.32–0.83	1.08 ± 0.83 (1.13)	0.67–1.48
M-CHX	0.45 ± 0.50 (0.50)	0.21–0.70	0.72 ± 0.83 (0.50)	0.31–1.12	1.17 ± 0.95 (1.13)	0.71–1.64
M-O	0.59 ± 0.80 (0.38)	0.20–0.98	0.28 ± 0.27 (0.25)	0.15–0.41	0.88 ± 0.74 (0.63)	0.51–1.24
M-GPA	0.70 ± 0.59 (0.50)	0.42–0.99	0.81 ± 0.63 (0.88)	0.50–1.12	1.52 ± 0.58 (1.63)	1.23–1.80
GPA	0.22 ± 0.54 (0.25)	–0.05–0.48	0.34 ± 0.51 (0.25)	0.10–0.59	0.56 ± 0.71 (0.75)	0.21–0.91
BoP (%)						
M	18.75 ± 45.18 (25)	-3.39–40.89	21.88 ± 34.00 (25)	5.21–38.54	40.62 ± 27.20 (50)	27.30–53.95
M-CHX	26.56 ± 30.91 (25)	11.41– 41.71	25.00 ± 28.87 (25)	10.85–39.15	51.56 ± 21.35 (50)	41.10–62.02
M-O	31.25 ± 39.26 25	12.01–50.49	7.81 ± 26.95 (0)	-5.40–21.02	39.06 ± 40.79 (50)	19.07–59.05
M-GPA	50.00 ± 25.82 (50)	37.35–62.65	23.44 ± 23.22 (25)	12.06–34.81	73.44 ± 28.09 (75)	59.67–87.20
GPA	14.06 ± 18.19 0	5.15–22.97	31.25 ± 35.94 (37.5)	13.64–48.86	45.31 ± 38.96 (50)	26.22–64.41
mPI						
M	0.33 ± 0.34 (0.38)	0.16–0.49	0.36 ± 0.39 (0.5)	0.170–0.55	0.69 ± 0.38 (0.75)	0.50–0.87
M-CHX	0.53 ± 0.31 (0.5)	0.38–0.69	0.12 ± 0.38 (0)	-0.06–0.31	0.66 ± 0.40 (0.5)	0.46–0.85
M-O	0.38 ± 0.35 (0.25)	0.20–0.55	0.31 ± 0.42 (0.13)	0.11–0.52	0.69 ± 0.38 (0.75)	0.50–0.87
M-GPA	0.67 ± 0.34 (0.75)	0.51–0.84	0.25 ± 0.50 (0.25)	0.01–0.49	0.92 ± 0.43 (1)	0.71–1.13
GPA	0.39 ± 0.48 (0.25)	0.15–0.63	0.28 ± 0.38 (0.25)	0.10–0.46	0.67 ± 0.49 (0.75)	0.43–0.91

Mean values ± standard deviations and medians of probing pocket depth (PPD, mm), bleeding on probing (BoP, %), and modified plaque index (mPI) are presented for each time point. CI: confidence interval.

### Probing pocket depth (PPD)

From T0 to T1, the smallest reduction occurred in group GPA (0.22 ± 0.54 mm) and the greatest in group M-GPA (0.70 ± 0.59 mm). However, the mixed-effects model demonstrated no statistically significant differences among groups (χ^2^ = 1.36, df = 4, *p* = 0.85).

Between T1 and T2, PPD reductions were maintained or further improved across all groups, with the largest reduction again seen in group M-GPA (0.81 ± 0.63 mm), yet intergroup differences remained nonsignificant (χ^2^ = 5.38, df = 4, *p* = 0.251).

Across the full 6-month period (T0–T2), group M-GPA achieved the most pronounced PPD reduction (1.52 ± 0.58 mm), followed by M-O and M-CHX, while the smallest reduction was recorded in group GPA (0.56 ± 0.71 mm). Despite these numerical differences, comparative analysis revealed no statistically significant intergroup differences (χ^2^ = 5.96, df = 4, *p* = 0.202).

### Bleeding on probing (BoP)

From T0 to T1, the greatest reduction occurred in group M-GPA (50.00 ± 25.82%), whereas group GPA showed the smallest decrease (14.06 ± 18.19%). Intergroup differences were not statistically significant (χ^2^ = 5.84, df = 4, *p* = 0.211).

Between T1 and T2, BoP remained stable or improved further, with group GPA showing the largest decrease (31.25 ± 35.94%) and group M-O the smallest (7.81 ± 26.95%). Differences were not significant (χ^2^ = 3.11, df = 4, *p* = 0.539).

Over the full study period (T0–T2), the most substantial BoP reduction was observed in group M-GPA (73.44 ± 28.09%), followed by M-CHX (51.56 ± 21.35%); however, these differences did not reach statistical significance (χ^2^ = 6.56, df = 4, *p* = 0.161).

### Modified plaque index (mPI)

From T0 to T1, the greatest reduction occurred in group M-GPA (0.67 ± 0.34) and the smallest in group M (0.33 ± 0.34), without significant intergroup differences (χ^2^ = 7.99, df = 4, *p* = 0.092).

Between T1 and T2, mPI levels remained relatively stable, with the largest reduction in group M (0.36 ± 0.39) and the smallest in M-CHX (0.12 ± 0.38); these differences were nonsignificant (χ^2^ = 2.64, df = 4, *p* = 0.619).

Across T0–T2, group M-GPA again showed the greatest mPI reduction (0.92 ± 0.43), whereas group GPA had the smallest (0.67 ± 0.49). Nonetheless, intergroup differences remained statistically nonsignificant (χ^2^ = 3.13, df = 4, *p* = 0.537).

### Treatment success

At 3 months (T1), the proportion of implants meeting the treatment success criteria (PPD ≤ 5 mm and BoP at ≤ 1 site) ranged from 0% in the GPA group to 56.3% in the M-GPA group. Mixed-effects logistic regression analysis revealed that only the M-GPA group achieved significantly higher odds of success compared with control group M (OR = 5.57, 95% CI 1.24–21.9; *p* = 0.01). At 6 months (T2), treatment success increased across all groups, with the highest observed success remaining in the M-GPA group (87.5%, model-estimated probability: 86.7%). The M-GPA group maintained a statistically significant advantage over the control group M (OR = 6.26, 95% CI 2.10–18.30; *p* < 0.01). Although the GPA group showed a notable improvement in success at 6 months (68.8%, 11/16), this difference did not reach statistical significance relative to control group M. Absolute numbers and model-based estimates are presented in Table 4.

**Table 4:** Implant-level treatment success (observed and model-estimated) at 3 months (T1) and 6 months (T2) in the treatment groups.

	T1				T2			
	Observed n/N (%)	Predicted %	OR vs M (95% CI)	*p*-value	Observed n/N (%)	Predicted %	OR vs M (95% CI)	*p*-value
M	3/16 (18.8)	18.8	Reference	–	8/16 (50.0)	50.8	Reference	–
M-CHX	2/16 (12.5)	12.5	0.62 (0.10–3.55)	0.59	10/16 (62.5)	62.6	1.62 (0.42–5.82)	0.32
M-O	5/16 (31.3)	31.3	1.97 (0.38–8.24)	0.22	9/16 (56.3)	56.3	1.25 (0.31–4.25)	0.44
M-GPA	9/16 (56.3)	56.3	5.57 (1.24–21.9)	0.01*	14/16 (87.5)	86.7	6.26 (2.10–18.3)	< 0.01*
GPA	0/16 (0.0)	~ 0	–	–	11/16 (68.8)	68.7	2.13 (0.61–7.06)	0.12

Treatment success was defined as PPD ≤ 5 mm and BoP present at ≤ 1 site per implant. Observed values are presented as n/N (%). Predicted probabilities, odds ratios (OR), and *p*-values were obtained from generalized linear mixed-effects models (logit link) with random intercepts for patients. *p < 0.05.

## Discussion

The aim of the present 6-month, assessor-blinded, parallel-arm clinical trial was to compare the efficacy of nonsurgical mechanical submarginal instrumentation alone with its combination with CHX, gaseous ozone, or glycine powder air abrasion, as well as with glycine powder air abrasion used as monotherapy, in the management of early-diagnosed peri-implantitis lesions. To the best of our knowledge, this is one of the first randomized multi-arm trials to evaluate three adjunctive modalities (CHX, ozone, glycine air abrasion) alongside glycine air abrasion as monotherapy under a standardized mechanical control. This design addresses a persistent gap in the peri-implantitis literature, where comprehensive head-to-head comparisons of adjunctive therapies remain scarce. Across the 6-month period, all treatment strategies produced improvements in PPD, BoP, and mPI; however, no statistically significant differences emerged between groups, despite numerically superior trends in the M-GPA arm.

Management of peri-implant diseases encompasses a broad spectrum of nonsurgical and surgical interventions. Although nonsurgical therapy effectively resolves peri-implant mucositis^24^, its predictability in peri-implantitis—particularly beyond the earliest stages—remains uncertain^25^. Numerous adjunctive modalities have been proposed to enhance nonsurgical outcomes, yet systematic reviews consistently report no clearly superior approach^18,19,26,27^, and recent clinical practice guidelines underscore the absence of a universally endorsed protocol supported by high-level evidence^6^. Given the established role of nonsurgical therapy as the first step in the stepwise management of peri-implantitis^28^, the present multi-arm design sought to determine whether adjunctive approaches confer meaningful clinical advantages over mechanical debridement alone^20^.

The study targeted implants with 4–6 mm probing depths and radiographic bone loss < 25% of implant length, consistent with the early peri-implantitis definition by Froum and Rosen^21^. As noted previously^1,2^ this early stage may overlap with peri-implant mucositis, especially when longitudinal radiographs are unavailable. To mitigate this risk, baseline radiographs were scrutinized to ensure inclusion of implants showing evidence of disease progression. While this approach increased diagnostic specificity, some inherent ambiguity at the mild end of the peri-implantitis spectrum cannot be entirely eliminated. Because current disease classifications do not stratify peri-implantitis severity^29^, the findings should be interpreted within the context of early inflammatory lesions with limited submarginal bone involvement. Although not generalizable to advanced peri-implantitis, the results offer insights into the conservative management of early disease. In the absence of comparable multi-arm RCTs with standardized controls^30^, interpretation is framed primarily against recent systematic reviews evaluating similar interventions.

Mechanical submarginal instrumentation alone (M group) produced significant reductions in PPD, BoP, and mPI, aligning with previously reported ranges (PPD: 0.2–1.8 mm; BoP: 5.3–57.1%)^26^. These improvements were not statistically different from those in the adjunctive groups, indicating that mechanical debridement alone can achieve comparable short-term outcomes in early peri-implantitis, as reported in earlier literature^17,19^.

Adjunctive CHX, despite its broad antimicrobial profile and substantivity^31^, did not yield superior outcomes compared with mechanical therapy alone. This aligns with prior RCTs showing limited incremental benefit^32,33^ and systematic reviews similarly concluding modest or negligible added value^18,34^. Variability in delivery protocols and concentrations may contribute to inconsistent findings.

Gaseous ozone demonstrated significant intragroup improvements consistent with its antimicrobial and oxidative effects^35,36^. However, these outcomes did not differ significantly from those of other modalities. This mirrors the mixed evidence reported in previous clinical trials^37–40^ and the conclusions of systematic reviews indicating insufficient evidence to support ozone as a routine adjunct in nonsurgical peri-implantitis therapy^41,42^.

Glycine powder air abrasion continues to attract interest due to its gentle yet effective biofilm-disrupting properties^43^. A recent RCT by Partido et al.^44^ demonstrated that glycine air abrasion promoted early and sustained reductions in inflammatory markers, accompanied by rapid shifts toward a health-compatible microbiome and favorable cytokine modulation during the initial 3 months of therapy. Although their study addressed mucositis rather than peri-implantitis, the findings offer mechanistic support for glycine-based debridement. In the present trial, adjunctive glycine air abrasion (M-GPA group) produced the greatest numerical improvements across all clinical parameters, whereas monotherapy yielded more modest gains. While these trends did not reach statistical significance, they are consistent with previous studies reporting variable but encouraging clinical responses^9,16,45–47^ and systematic reviews underscoring the need for further RCTs to clarify its role^14,17,48,49^. Although statistical superiority was not demonstrated, glycine air abrasion may enhance soft-tissue inflammation control and contribute to more favorable clinical trajectories during early peri-implant disease management.

Despite varying degrees of improvement across groups, the lack of statistically significant intergroup differences highlights the ongoing challenge of establishing superiority among nonsurgical peri-implantitis therapies. Nonetheless, these findings may inform clinical decision-making in early lesions, particularly where diagnostic overlap with mucositis exists. Mechanical instrumentation—alone or combined with adjunctive CHX, ozone, or glycine air abrasion—can provide meaningful short-term improvements.

This study has limitations. Residual intra-subject correlations may persist despite mixed-effects modeling; the sample size and follow-up duration limit generalizability; and the absence of microbiological or radiographic reassessment restricts interpretation to soft-tissue parameters. The lack of composite treatment-success criteria and PROMs narrows the assessment of clinical impact. Additionally, the original sample-size calculation did not account for clustering or multi-arm multiplicity, raising the possibility of underpowering. Finally, the random-effects structure did not incorporate an implant-level intercept, potentially limiting the ability to capture intra-implant variability.

In conclusion, all nonsurgical modalities evaluated in this trial yielded clinically meaningful improvements in early peri-implant lesions, underscoring the central role of mechanical debridement. Although adjunctive glycine powder air abrasion produced the most substantial numerical gains, none of the tested approaches demonstrated statistical superiority. These findings reinforce mechanical instrumentation as the cornerstone of early-stage peri-implantitis care while highlighting the potential complementary value but not proven superiority of adjunctive therapies.

## Conclusion

Overall, within the limitations of this study, conventional mechanical instrumentation remains the cornerstone of nonsurgical peri-implantitis therapy, providing significant improvements in clinical parameters over 6 months. Although no statistically significant differences were detected among treatment groups, the adjunctive use of glycine powder air abrasion produced the most pronounced numerical improvements, suggesting a potential role in enhancing inflammation control at the clinical level. Future studies with larger sample sizes, longer follow-up, and additional outcome measures are needed to clarify the effectiveness of adjunctive approaches compared to conventional mechanical therapy.

## Data Availability

The datasets generated and/or analyzed during the current study are available from the corresponding author upon reasonable request.
